# Supplementary material for: Genome-Wide Identification and Expression Analysis of WRKY Transcription Factors in Siraitia siamensis
Source: Plants (Basel). 2023 Jan 7;12(2):288. doi: 10.3390/plants12020288 (PMC9861706; doi:10.3390/plants12020288)
Supplement: Supplementary file 1 [file plants-12-00288-s001.zip › plants-2083743-supplementary.pdf]

Table S1. The details of 10 motifs in the protein sequences of SsWRKYs.

Domain	E-value	Sites	Width	Multilevel consensus sequence
1	$1.4e^{-1318}$	59	29	ILDDGYRWRKYGQKPVKGSPYPRSYYRCT
2	$2.1e^{-1017}$	59	29	GCPVRKQVZRSSEDPSIVITTYEGEHNHP
3	$6.6e^{-189}$	10	30	EDGYNWRKYGQKQVKGSEYPRSYYKCTHPN
4	$4.6e^{-155}$	24	21	KKGEKRVREPRFAFQTKSEVD
5	$1.02e^{-87}$	10	24	LDGQITEIIYKGQHNHPKPQPNRR
6	$2.3e^{-51}$	9	38	LQAELERINTENQRLKDMLNQVTSNYHALQMHFTTLIQ
7	$2.0e^{-49}$	17	15	KKRKNRVKRVVRVPA
8	$3.5e^{-31}$	8	21	CEAVTDAAVSKFKKVISLLNR
9	$2.7e^{-33}$	7	29	DTLSAATAAITADPNFTAALAAAISSIIG
10	$4.8e^{-30}$	8	23	PSPYFTIPPGLSPTELLDSPVLL

Table S2. The collinear analysis of *WRKY* genes between *S. siamensis* and *A. thaliana*, *O. sativa*, and *C. sativus*, respectively.

Gene_ID	Ss_Chrom	Gene_name	Gene_ID	Chrom	Gene_name	Species	Type
SsiaChr1G00023090.1	Ssia_chr1	*SsWRKY2*	AT1G13960.1.TAIR10	Chr1	*AtWRKY4*	*Arabidopsis thaliana*	I
SsiaChr1G00040860.1	Ssia_chr1	*SsWRKY3*	AT2G04880.1.TAIR10	Chr2	*AtWRKY1*	*Arabidopsis thaliana*	I
SsiaChr1G00023090.1	Ssia_chr1	*SsWRKY2*	AT2G03340.1.TAIR10	Chr2	*AtWRKY3*	*Arabidopsis thaliana*	I
SsiaChr1G00023090.1	Ssia_chr1	*SsWRKY2*	AT3G01080.1.TAIR10	Chr3	*AtWRKY58*	*Arabidopsis thaliana*	I
SsiaChr1G00020800.1	Ssia_chr1	*SsWRKY1*	AT3G01970.1.TAIR10	Chr3	*AtWRKY45*	*Arabidopsis thaliana*	IIc
SsiaChr1G00042810.1	Ssia_chr1	*SsWRKY5*	AT3G58710.1.TAIR10	Chr3	*AtWRKY69*	*Arabidopsis thaliana*	IIe
SsiaChr2G00004890.1	Ssia_chr2	*SsWRKY8*	AT1G80840.1.TAIR10	Chr1	*AtWRKY40*	*Arabidopsis thaliana*	IIa
SsiaChr2G00001280.1	Ssia_chr2	*SsWRKY7*	AT1G69310.1.TAIR10	Chr1	*AtWRKY57*	*Arabidopsis thaliana*	IIc
SsiaChr2G00005450.1	Ssia_chr2	*SsWRKY10*	AT5G26170.1.TAIR10	Chr5	*AtWRKY50*	*Arabidopsis thaliana*	IIc
SsiaChr3G00056180.1	Ssia_chr3	*SsWRKY15*	AT2G47260.1.TAIR10	Chr2	*AtWRKY23*	*Arabidopsis thaliana*	IIc
SsiaChr3G00046440.1	Ssia_chr3	*SsWRKY13*	AT2G37260.1.TAIR10	Chr2	*AtWRKY44*	*Arabidopsis thaliana*	I
SsiaChr3G00044550.1	Ssia_chr3	*SsWRKY12*	AT2G38470.1.TAIR10	Chr2	*AtWRKY33*	*Arabidopsis thaliana*	I
SsiaChr3G00062240.1	Ssia_chr3	*SsWRKY16*	AT4G23550.1.TAIR10	Chr4	*AtWRKY29*	*Arabidopsis thaliana*	IIe

SsiaChr3G00062690.1	Ssia_chr3	*SsWRKY17*	AT4G23810.1.TAIR10	Chr4	*AtWRKY53*	*Arabidopsis thaliana*	III
SsiaChr3G00062240.1	Ssia_chr3	*SsWRKY16*	AT4G01250.1.TAIR10	Chr4	*AtWRKY22*	*Arabidopsis thaliana*	IIe
SsiaChr3G00056180.1	Ssia_chr3	*SsWRKY15*	AT5G49520.1.TAIR10	Chr5	*AtWRKY48*	*Arabidopsis thaliana*	IIc
SsiaChr3G00044220.1	Ssia_chr3	*SsWRKY11*	AT5G01900.1.TAIR10	Chr5	*AtWRKY62*	*Arabidopsis thaliana*	III
SsiaChr3G00054430.1	Ssia_chr3	*SsWRKY14*	AT5G13080.1.TAIR10	Chr5	*AtWRKY75*	*Arabidopsis thaliana*	IIc
SsiaChr4G00085620.1	Ssia_chr4	*SsWRKY21*	AT1G62300.1.TAIR10	Chr1	*AtWRKY6*	*Arabidopsis thaliana*	IIb
SsiaChr4G00082080.1	Ssia_chr4	*SsWRKY19*	AT1G30650.1.TAIR10	Chr1	*AtWRKY14*	*Arabidopsis thaliana*	IIe
SsiaChr4G00083060.1	Ssia_chr4	*SsWRKY20*	AT1G29860.1.TAIR10	Chr1	*AtWRKY71*	*Arabidopsis thaliana*	IIc
SsiaChr4G00090590.1	Ssia_chr4	*SsWRKY22*	AT2G23320.1.TAIR10	Chr2	*AtWRKY15*	*Arabidopsis thaliana*	IId
SsiaChr4G00082080.1	Ssia_chr4	*SsWRKY19*	AT2G34830.1.TAIR10	Chr2	*AtWRKY35*	*Arabidopsis thaliana*	IIe
SsiaChr4G00085620.1	Ssia_chr4	*SsWRKY21*	AT4G22070.1.TAIR10	Chr4	*AtWRKY31*	*Arabidopsis thaliana*	IIb
SsiaChr4G00085620.1	Ssia_chr4	*SsWRKY21*	AT4G04450.1.TAIR10	Chr4	*AtWRKY42*	*Arabidopsis thaliana*	IIb
SsiaChr4G00083060.1	Ssia_chr4	*SsWRKY20*	AT4G18170.1.TAIR10	Chr4	*AtWRKY28*	*Arabidopsis thaliana*	IIc
SsiaChr4G00091770.1	Ssia_chr4	*SsWRKY23*	AT4G39410.1.TAIR10	Chr4	*AtWRKY13*	*Arabidopsis thaliana*	IIc
SsiaChr4G00083060.1	Ssia_chr4	*SsWRKY20*	AT5G46350.1.TAIR10	Chr5	*AtWRKY8*	*Arabidopsis thaliana*	IIc
SsiaChr4G00082080.1	Ssia_chr4	*SsWRKY19*	AT5G45050.1.TAIR10	Chr5	*AtWRKY16*	*Arabidopsis thaliana*	IIe
SsiaChr5G00107540.1	Ssia_chr5	*SsWRKY26*	AT1G80840.1.TAIR10	Chr1	*AtWRKY40*	*Arabidopsis thaliana*	IIa
SsiaChr5G00108780.1	Ssia_chr5	*SsWRKY27*	AT2G24570.1.TAIR10	Chr2	*AtWRKY17*	*Arabidopsis thaliana*	IId
SsiaChr5G00105720.1	Ssia_chr5	*SsWRKY25*	AT2G23320.1.TAIR10	Chr2	*AtWRKY15*	*Arabidopsis thaliana*	IId
SsiaChr5G00108780.1	Ssia_chr5	*SsWRKY27*	AT4G31550.1.TAIR10	Chr4	*AtWRKY11*	*Arabidopsis thaliana*	IId
SsiaChr5G00107540.1	Ssia_chr5	*SsWRKY26*	AT4G31800.1.TAIR10	Chr4	*AtWRKY18*	*Arabidopsis thaliana*	IIa
SsiaChr6G00152850.1	Ssia_chr6	*SsWRKY28*	AT2G30250.1.TAIR10	Chr2	*AtWRKY25*	*Arabidopsis thaliana*	I
SsiaChr6G00159600.1	Ssia_chr6	*SsWRKY30*	AT2G23320.1.TAIR10	Chr2	*AtWRKY15*	*Arabidopsis thaliana*	IId
SsiaChr7G00073400.1	Ssia_chr7	*SsWRKY35*	AT1G64000.1.TAIR10	Chr1	*AtWRKY56*	*Arabidopsis thaliana*	IIc
SsiaChr7G00080620.1	Ssia_chr7	*SsWRKY38*	AT2G46400.1.TAIR10	Chr2	*AtWRKY46*	*Arabidopsis thaliana*	III
SsiaChr7G00073400.1	Ssia_chr7	*SsWRKY35*	AT2G46130.1.TAIR10	Chr2	*AtWRKY43*	*Arabidopsis thaliana*	IIc
SsiaChr7G00073090.1	Ssia_chr7	*SsWRKY34*	AT2G46400.1.TAIR10	Chr2	*AtWRKY46*	*Arabidopsis thaliana*	III
SsiaChr7G00073090.1	Ssia_chr7	*SsWRKY34*	AT4G11070.1.TAIR10	Chr4	*AtWRKY41*	*Arabidopsis thaliana*	III
SsiaChr7G00073410.1	Ssia_chr7	*SsWRKY36*	AT4G23550.1.TAIR10	Chr4	*AtWRKY29*	*Arabidopsis thaliana*	IIe
SsiaChr7G00073090.1	Ssia_chr7	*SsWRKY34*	AT4G23810.1.TAIR10	Chr4	*AtWRKY53*	*Arabidopsis thaliana*	III

SsiaChr7G00080620.1	Ssia_chr7	*SsWRKY38*	AT5G24110.1.TAIR10	Chr5	*AtWRKY30*	*Arabidopsis thaliana*	III
SsiaChr7G00068370.1	Ssia_chr7	*SsWRKY33*	AT5G56270.1.TAIR10	Chr5	*AtWRKY2*	*Arabidopsis thaliana*	I
SsiaChr7G00073400.1	Ssia_chr7	*SsWRKY35*	AT5G41570.1.TAIR10	Chr5	*AtWRKY24*	*Arabidopsis thaliana*	IIc
SsiaChr7G00073410.1	Ssia_chr7	*SsWRKY36*	AT5G52830.1.TAIR10	Chr5	*AtWRKY27*	*Arabidopsis thaliana*	IIe
SsiaChr8G00134260.1	Ssia_chr8	*SsWRKY42*	AT1G18860.1.TAIR10	Chr1	*AtWRKY61*	*Arabidopsis thaliana*	IIb
SsiaChr8G00134260.1	Ssia_chr8	*SsWRKY42*	AT1G69810.1.TAIR10	Chr1	*AtWRKY36*	*Arabidopsis thaliana*	IIb
SsiaChr8G00130410.1	Ssia_chr8	*SsWRKY40*	AT4G30935.1.TAIR10	Chr4	*AtWRKY32*	*Arabidopsis thaliana*	I
SsiaChr9G00184810.1	Ssia_chr9	*SsWRKY44*	AT1G30650.1.TAIR10	Chr1	*AtWRKY14*	*Arabidopsis thaliana*	IIe
SsiaChr9G00186390.1	Ssia_chr9	*SsWRKY45*	AT1G29280.1.TAIR10	Chr1	*AtWRKY65*	*Arabidopsis thaliana*	IIe
SsiaChr9G00190810.1	Ssia_chr9	*SsWRKY46*	AT1G18860.1.TAIR10	Chr1	*AtWRKY61*	*Arabidopsis thaliana*	IIb
SsiaChr9G00178500.1	Ssia_chr9	*SsWRKY43*	AT2G30250.1.TAIR10	Chr2	*AtWRKY25*	*Arabidopsis thaliana*	I
SsiaChr9G00184810.1	Ssia_chr9	*SsWRKY44*	AT2G34830.1.TAIR10	Chr2	*AtWRKY35*	*Arabidopsis thaliana*	IIe
SsiaChr9G00184810.1	Ssia_chr9	*SsWRKY44*	AT5G45260.1.TAIR10	Chr5	*AtWRKY52*	*Arabidopsis thaliana*	IIe
SsiaChr10G00175160.1	Ssia_chr10	*SsWRKY49*	AT1G13960.1.TAIR10	Chr1	*AtWRKY4*	*Arabidopsis thaliana*	I
SsiaChr10G00168460.1	Ssia_chr10	*SsWRKY48*	AT2G40740.1.TAIR10	Chr2	*AtWRKY55*	*Arabidopsis thaliana*	III
SsiaChr10G00168450.1	Ssia_chr10	*SsWRKY47*	AT2G40750.1.TAIR10	Chr2	*AtWRKY54*	*Arabidopsis thaliana*	III
SsiaChr10G00175160.1	Ssia_chr10	*SsWRKY49*	AT2G03340.1.TAIR10	Chr2	*AtWRKY3*	*Arabidopsis thaliana*	I
SsiaChr10G00168450.1	cSsia_hr10	*SsWRKY47*	AT3G56400.1.TAIR10	Chr3	*AtWRKY70*	*Arabidopsis thaliana*	III
SsiaChr11G00223210.1	Ssia_chr11	*SsWRKY53*	AT1G80840.1.TAIR10	Chr1	*AtWRKY40*	*Arabidopsis thaliana*	IIa
SsiaChr11G00221950.1	Ssia_chr11	*SsWRKY52*	AT2G47260.1.TAIR10	Chr2	*AtWRKY23*	*Arabidopsis thaliana*	IIc
SsiaChr11G00215290.1	Ssia_chr11	*SsWRKY50*	AT3G01970.1.TAIR10	Chr3	*AtWRKY45*	*Arabidopsis thaliana*	IIc
SsiaChr11G00221950.1	Ssia_chr11	*SsWRKY52*	AT3G62340.1.TAIR10	Chr3	*AtWRKY68*	*Arabidopsis thaliana*	IIc
SsiaChr12G00195640.1	Ssia_chr12	*SsWRKY54*	AT2G46400.1.TAIR10	Chr2	*AtWRKY46*	*Arabidopsis thaliana*	III
SsiaChr12G00203270.1	Ssia_chr12	*SsWRKY55*	AT3G62340.1.TAIR10	Chr3	*AtWRKY68*	*Arabidopsis thaliana*	IIc
SsiaChr12G00203270.1	Ssia_chr12	*SsWRKY55*	AT5G49520.1.TAIR10	Chr5	*AtWRKY48*	*Arabidopsis thaliana*	IIc
SsiaChr13G00142910.1	Ssia_chr13	*SsWRKY57*	AT2G47260.1.TAIR10	Chr2	*AtWRKY23*	*Arabidopsis thaliana*	IIc
SsiaChr13G00142910.1	Ssia_chr13	*SsWRKY57*	AT3G62340.1.TAIR10	Chr3	*AtWRKY68*	*Arabidopsis thaliana*	IIc
SsiaChr13G00142910.1	Ssia_chr13	*SsWRKY57*	AT5G49520.1.TAIR10	Chr5	*AtWRKY48*	*Arabidopsis thaliana*	IIc
SsiaChr14G00213550.1	Ssia_chr14	*SsWRKY59*	AT5G52830.1.TAIR10	Chr5	*AtWRKY27*	*Arabidopsis thaliana*	IIe
SsiaChr2G00004890.1	Ssia_chr2	*SsWRKY8*	PAC:24129555	Chr2	-	*Oryza sativa*	IIa

SsiaChr2G00001280.1	Ssia_chr2	*SsWRKY7*	PAC:24125535	Chr3	-	*Oryza sativa*	IIc
SsiaChr2G00004890.1	Ssia_chr2	*SsWRKY8*	PAC:24135826	Chr9	-	*Oryza sativa*	IIa
SsiaChr3G00044550.1	Ssia_chr3	*SsWRKY12*	PAC:24121503	Chr1	-	*Oryza sativa*	I
SsiaChr3G00062240.1	Ssia_chr3	*SsWRKY16*	PAC:24117685	Chr1	-	*Oryza sativa*	IIe
SsiaChr3G00044220.1	Ssia_chr3	*SsWRKY11*	PAC:24159224	Chr11	-	*Oryza sativa*	III
SsiaChr3G00044220.1	Ssia_chr3	*SsWRKY11*	PAC:24145659	Chr12	-	*Oryza sativa*	III
SsiaChr3G00062240.1	Ssia_chr3	*SsWRKY16*	PAC:24154354	Chr5	-	*Oryza sativa*	IIe
SsiaChr3G00044550.1	Ssia_chr3	*SsWRKY12*	PAC:24154429	Chr5	-	*Oryza sativa*	I
SsiaChr4G00095300.1	Ssia_chr4	*SsWRKY24*	PAC:24117010	Chr1	-	*Oryza sativa*	III
SsiaChr5G00107540.1	Ssia_chr5	*SsWRKY26*	PAC:24129555	Chr2	-	*Oryza sativa*	IIa
SsiaChr5G00107540.1	Ssia_chr5	*SsWRKY26*	PAC:24141799	Chr6	-	*Oryza sativa*	IIa
SsiaChr7G00073410.1	Ssia_chr7	*SsWRKY36*	PAC:24117685	Chr1	-	*Oryza sativa*	IIe
SsiaChr7G00080620.1	Ssia_chr7	*SsWRKY38*	PAC:24118091	Chr1	-	*Oryza sativa*	III
SsiaChr7G00073410.1	Ssia_chr7	*SsWRKY36*	PAC:24154354	Chr5	-	*Oryza sativa*	IIe
SsiaChr8G00131810.1	Ssia_chr8	*SsWRKY41*	PAC:24104755	Chr4	-	*Oryza sativa*	IId
SsiaChr8G00130410.1	Ssia_chr8	*SsWRKY40*	PAC:24101198	Chr8	-	*Oryza sativa*	I
SsiaChr9G00178500.1	Ssia_chr9	*SsWRKY43*	PAC:24121503	Chr1	-	*Oryza sativa*	I
SsiaChr9G00186390.1	Ssia_chr9	*SsWRKY45*	PAC:24118401	Chr1	-	*Oryza sativa*	IIe
SsiaChr9G00178500.1	Ssia_chr9	*SsWRKY43*	PAC:24154429	Chr5	-	*Oryza sativa*	I
SsiaChr10G00168450.1	Ssia_chr10	*SsWRKY47*	PAC:24159224	Chr11	-	*Oryza sativa*	III
SsiaChr10G00168450.1	Ssia_chr10	*SsWRKY47*	PAC:24145659	Chr12	-	*Oryza sativa*	III
SsiaChr10G00175160.1	Ssia_chr10	*SsWRKY49*	PAC:24125917	Chr3	-	*Oryza sativa*	I
SsiaChr10G00175160.1	Ssia_chr10	*SsWRKY49*	PAC:24113117	Chr7	-	*Oryza sativa*	I
SsiaChr11G00223210.1	Ssia_chr11	*SsWRKY53*	PAC:24141799	Chr6	-	*Oryza sativa*	IIa
SsiaChr13G00140450.1	Ssia_chr13	*SsWRKY56*	PAC:24148383	Chr12	-	*Oryza sativa*	IIe
SsiaChr13G00140450.1	Ssia_chr13	*SsWRKY56*	PAC:24122325	Chr3	-	*Oryza sativa*	IIe
SsiaChr14G00204380.1	Ssia_chr14	*SsWRKY58*	PAC:24128365	Chr3	-	*Oryza sativa*	IIe
SsiaChr14G00204380.1	Ssia_chr14	*SsWRKY58*	PAC:24122325	Chr3	-	*Oryza sativa*	IIe
SsiaChr1G00042810.1	Ssia_chr1	*SsWRKY5*	transcript:KGN65607	Chr1	-	*Cucumis sativus*	IIe
SsiaChr1G00020800.1	Ssia_chr1	*SsWRKY1*	transcript:KGN63156	Chr2	-	*Cucumis sativus*	IIc

SsiaChr1G00023090.1	Ssia_chr1	*SsWRKY2*	transcript:KGN52490	Chr5	-	*Cucumis sativus*	I
SsiaChr1G00023090.1	Ssia_chr1	*SsWRKY2*	transcript:KGN51956	Chr5	-	*Cucumis sativus*	I
SsiaChr1G00041580.1	Ssia_chr1	*SsWRKY4*	transcript:KGN49089	Chr6	-	*Cucumis sativus*	IIb
SsiaChr1G00043600.1	Ssia_chr1	*SsWRKY6*	transcript:KGN46828	Chr6	-	*Cucumis sativus*	IIc
SsiaChr1G00040860.1	Ssia_chr1	*SsWRKY3*	transcript:KGN43427	Chr7	-	*Cucumis sativus*	I
SsiaChr1G00041580.1	Ssia_chr1	*SsWRKY4*	transcript:KGN43509	Chr7	-	*Cucumis sativus*	IIb
SsiaChr1G00043600.1	Ssia_chr1	*SsWRKY6*	transcript:KGN44260	Chr7	-	*Cucumis sativus*	IIc
SsiaChr1G00042810.1	Ssia_chr1	*SsWRKY5*	transcript:KGN43635	Chr7	-	*Cucumis sativus*	IIa
SsiaChr2G00004890.1	Ssia_chr2	*SsWRKY8*	transcript:KGN62992	Chr2	-	*Cucumis sativus*	IIa
SsiaChr2G00004890.1	Ssia_chr2	*SsWRKY8*	transcript:KGN55434	Chr4	-	*Cucumis sativus*	IIa
SsiaChr2G00001280.1	Ssia_chr2	*SsWRKY7*	transcript:KGN47639	Chr6	-	*Cucumis sativus*	IIc
SsiaChr2G00005450.1	Ssia_chr2	*SsWRKY10*	transcript:KGN47600	Chr6	-	*Cucumis sativus*	IIc
SsiaChr3G00044220.1	Ssia_chr3	*SsWRKY11*	transcript:KGN58681	Chr3	-	*Cucumis sativus*	III
SsiaChr3G00044550.1	Ssia_chr3	*SsWRKY12*	transcript:KGN58716	Chr3	-	*Cucumis sativus*	I
SsiaChr3G00046440.1	Ssia_chr3	*SsWRKY13*	transcript:KGN58924	Chr3	-	*Cucumis sativus*	I
SsiaChr3G00063680.1	Ssia_chr3	*SsWRKY18*	transcript:KGN56333	Chr3	-	*Cucumis sativus*	IIc
SsiaChr3G00062690.1	Ssia_chr3	*SsWRKY17*	transcript:KGN56437	Chr3	-	*Cucumis sativus*	III
SsiaChr3G00062240.1	Ssia_chr3	*SsWRKY16*	transcript:KGN56482	Chr3	-	*Cucumis sativus*	IIe
SsiaChr3G00056180.1	Ssia_chr3	*SsWRKY15*	transcript:KGN57179	Chr3	-	*Cucumis sativus*	IIc
SsiaChr3G00062690.1	Ssia_chr3	*SsWRKY17*	transcript:KGN57857	Chr3	-	*Cucumis sativus*	III
SsiaChr3G00054430.1	Ssia_chr3	*SsWRKY14*	transcript:KGN53043	Chr4	-	*Cucumis sativus*	IIc
SsiaChr3G00062690.1	Ssia_chr3	*SsWRKY17*	transcript:KGN53395	Chr4	-	*Cucumis sativus*	III
SsiaChr3G00062240.1	Ssia_chr3	*SsWRKY16*	transcript:KGN49916	Chr5	-	*Cucumis sativus*	IIe
SsiaChr4G00091770.1	Ssia_chr4	*SsWRKY23*	transcript:KGN62098	Chr2	-	*Cucumis sativus*	IIc
SsiaChr4G00095300.1	Ssia_chr4	*SsWRKY24*	transcript:KGN54262	Chr4	-	*Cucumis sativus*	III
SsiaChr4G00090590.1	Ssia_chr4	*SsWRKY22*	transcript:KGN55218	Chr4	-	*Cucumis sativus*	IId
SsiaChr4G00091770.1	Ssia_chr4	*SsWRKY23*	transcript:KGN48415	Chr6	-	*Cucumis sativus*	IIc
SsiaChr4G00090590.1	Ssia_chr4	*SsWRKY22*	transcript:KGN48545	Chr6	-	*Cucumis sativus*	IId
SsiaChr4G00085620.1	Ssia_chr4	*SsWRKY21*	transcript:KGN49089	Chr6	-	*Cucumis sativus*	IIb
SsiaChr4G00083060.1	Ssia_chr4	*SsWRKY20*	transcript:KGN49371	Chr6	-	*Cucumis sativus*	IIc

SsiaChr4G00082080.1	Ssia_chr4	*SsWRKY19*	transcript:KGN49489	Chr6	-	*Cucumis sativus*	IIe
SsiaChr4G00085620.1	Ssia_chr4	*SsWRKY21*	transcript:KGN43509	Chr7	-	*Cucumis sativus*	IIb
SsiaChr5G00107540.1	Ssia_chr5	*SsWRKY26*	transcript:KGN62992	Chr2	-	*Cucumis sativus*	IIa
SsiaChr5G00108780.1	Ssia_chr5	*SsWRKY27*	transcript:KGN60174	Chr3	-	*Cucumis sativus*	IId
SsiaChr5G00107540.1	Ssia_chr5	*SsWRKY26*	transcript:KGN55434	Chr4	-	*Cucumis sativus*	IIa
SsiaChr5G00105720.1	Ssia_chr5	*SsWRKY25*	transcript:KGN55218	Chr4	-	*Cucumis sativus*	IId
SsiaChr5G00105720.1	Ssia_chr5	*SsWRKY25*	transcript:KGN48545	Chr6	-	*Cucumis sativus*	IId
SsiaChr6G00159600.1	Ssia_chr6	*SsWRKY30*	transcript:KGN63315	Chr2	-	*Cucumis sativus*	IId
SsiaChr6G00161490.1	Ssia_chr6	*SsWRKY31*	transcript:KGN62098	Chr2	-	*Cucumis sativus*	IIc
SsiaChr6G00153650.1	Ssia_chr6	*SsWRKY29*	transcript:KGN46828	Chr6	-	*Cucumis sativus*	IIc
SsiaChr6G00159600.1	Ssia_chr6	*SsWRKY30*	transcript:KGN48545	Chr6	-	*Cucumis sativus*	IId
SsiaChr6G00152850.1	Ssia_chr6	*SsWRKY28*	transcript:KGN47228	Chr6	-	*Cucumis sativus*	I
SsiaChr6G00153650.1	Ssia_chr6	*SsWRKY29*	transcript:KGN44260	Chr7	-	*Cucumis sativus*	IIc
SsiaChr7G00067510.1	Ssia_chr7	*SsWRKY32*	transcript:KGN55986	Chr3	-	*Cucumis sativus*	I
SsiaChr7G00068370.1	Ssia_chr7	*SsWRKY33*	transcript:KGN56053	Chr3	-	*Cucumis sativus*	I
SsiaChr7G00073090.1	Ssia_chr7	*SsWRKY34*	transcript:KGN56437	Chr3	-	*Cucumis sativus*	III
SsiaChr7G00073400.1	Ssia_chr7	*SsWRKY35*	transcript:KGN56479	Chr3	-	*Cucumis sativus*	IIc
SsiaChr7G00073410.1	Ssia_chr7	*SsWRKY36*	transcript:KGN56482	Chr3	-	*Cucumis sativus*	IIe
SsiaChr7G00076520.1	Ssia_chr7	*SsWRKY37*	transcript:KGN57556	Chr3	-	*Cucumis sativus*	IIb
SsiaChr7G00080620.1	Ssia_chr7	*SsWRKY38*	transcript:KGN57857	Chr3	-	*Cucumis sativus*	III
SsiaChr7G00080620.1	Ssia_chr7	*SsWRKY38*	transcript:KGN56437	Chr3	-	*Cucumis sativus*	III
SsiaChr7G00080620.1	Ssia_chr7	*SsWRKY38*	transcript:KGN53395	Chr4	-	*Cucumis sativus*	III
SsiaChr7G00073410.1	Ssia_chr7	*SsWRKY36*	transcript:KGN53355	Chr4	-	*Cucumis sativus*	IIe
SsiaChr7G00073400.1	Ssia_chr7	*SsWRKY35*	transcript:KGN53356	Chr4	-	*Cucumis sativus*	IIc
SsiaChr7G00073090.1	Ssia_chr7	*SsWRKY34*	transcript:KGN53395	Chr4	-	*Cucumis sativus*	III
SsiaChr7G00073410.1	Ssia_chr7	*SsWRKY36*	transcript:KGN49916	Chr5	-	*Cucumis sativus*	IIe
SsiaChr8G00130410.1	Ssia_chr8	*SsWRKY40*	transcript:KGN65161	Chr1	-	*Cucumis sativus*	I
SsiaChr8G00134260.1	Ssia_chr8	*SsWRKY42*	transcript:KGN64250	Chr1	-	*Cucumis sativus*	IIb
SsiaChr8G00123950.1	Ssia_chr8	*SsWRKY39*	transcript:KGN66672	Chr1	-	*Cucumis sativus*	I
SsiaChr8G00134260.1	Ssia_chr8	*SsWRKY42*	transcript:KGN63654	Chr1	-	*Cucumis sativus*	IIb

SsiaChr9G00186390.1	Ssia_chr9	*SsWRKY45*	transcript:KGN65607	Chr1	-	*Cucumis sativus*	IIb
SsiaChr9G00190810.1	Ssia_chr9	*SsWRKY46*	transcript:KGN63654	Chr1	-	*Cucumis sativus*	IIb
SsiaChr9G00190810.1	Ssia_chr9	*SsWRKY46*	transcript:KGN64250	Chr1	-	*Cucumis sativus*	IIb
SsiaChr9G00184810.1	Ssia_chr9	*SsWRKY44*	transcript:KGN65962	Chr1	-	*Cucumis sativus*	IIe
SsiaChr9G00184810.1	Ssia_chr9	*SsWRKY44*	transcript:KGN49489	Chr6	-	*Cucumis sativus*	IIe
SsiaChr9G00186390.1	Ssia_chr9	*SsWRKY45*	transcript:KGN43635	Chr7	-	*Cucumis sativus*	IIb
SsiaChr10G00175160.1	Ssia_chr10	*SsWRKY49*	transcript:KGN51956	Chr5	-	*Cucumis sativus*	I
SsiaChr10G00168460.1	Ssia_chr10	*SsWRKY48*	transcript:KGN50746	Chr5	-	*Cucumis sativus*	III
SsiaChr10G00168450.1	Ssia_chr10	*SsWRKY47*	transcript:KGN50748	Chr5	-	*Cucumis sativus*	III
SsiaChr10G00175160.1	Ssia_chr10	*SsWRKY49*	transcript:KGN52490	Chr5	-	*Cucumis sativus*	I
SsiaChr11G00223210.1	Ssia_chr11	*SsWRKY53*	transcript:KGN58660	Chr3	-	*Cucumis sativus*	IIa
SsiaChr11G00215290.1	Ssia_chr11	*SsWRKY50*	transcript:KGN58164	Chr3	-	*Cucumis sativus*	IIc
SsiaChr11G00221880.1	Ssia_chr11	*SsWRKY51*	transcript:KGN52859	Chr4	-	*Cucumis sativus*	IIe
SsiaChr11G00223210.1	Ssia_chr11	*SsWRKY53*	transcript:KGN55434	Chr4	-	*Cucumis sativus*	IIa
SsiaChr11G00221950.1	Ssia_chr11	*SsWRKY52*	transcript:KGN45029	Chr7	-	*Cucumis sativus*	IIc
SsiaChr12G00203270.1	Ssia_chr12	*SsWRKY55*	transcript:KGN57179	Chr3	-	*Cucumis sativus*	IIc
SsiaChr12G00203270.1	Ssia_chr12	*SsWRKY55*	transcript:KGN47453	Chr6	-	*Cucumis sativus*	IIc
SsiaChr12G00195640.1	Ssia_chr12	*SsWRKY54*	transcript:KGN44546	Chr7	-	*Cucumis sativus*	III
SsiaChr13G00140450.1	Ssia_chr13	*SsWRKY56*	transcript:KGN44752	Chr7	-	*Cucumis sativus*	IIe
SsiaChr13G00142910.1	Ssia_chr13	*SsWRKY57*	transcript:KGN45029	Chr7	-	*Cucumis sativus*	IIc
SsiaChr14G00204380.1	Ssia_chr14	*SsWRKY58*	transcript:KGN61686	Chr2	-	*Cucumis sativus*	IIe
SsiaChr14G00213550.1	Ssia_chr14	*SsWRKY59*	transcript:KGN56482	Chr3	-	*Cucumis sativus*	IIe
SsiaChr14G00213550.1	Ssia_chr14	*SsWRKY59*	transcript:KGN49916	Chr5	-	*Cucumis sativus*	IIe

Table S3. The primers used for expression analysis for 15 *SsWRKY*s under cold stress.

Gene_name	5'primer	3'primer	Product size
SsWRKY12	AGTCTCTTCAACCTCCTCCATTC	GCTGCTCACCATCACAATCATT	174
SsWRKY13	ATGATGGGTATAACTGGCGGAAA	TGAGGCTTTGGGTGGTTGT	178
SsWRKY16	GCGTCTCCGATATGATTGTGAAC	CGGTGGCGGCATTGTTAA	138
SsWRKY20	TGCTTCGTTCTCGTCTTCTGAT	TGGTTCTCTGCTGCGTCTT	83

SsWRKY29	GATGGATACAGATGGAGAA	ACGCCTTGATACAGATAG	118
SsWRKY30	CCGCCGACTCTGAATCCAA	GAGCCACACTTCCTCTTGAGA	109
SsWRKY41	TCTCCTCCTGATGCCTCTGA	AGCCCTACTATCTGACGATTTGG	156
SsWRKY42	CCTCAACTTCTTCTTCCTCTCCTT	ATTGCCATAGCCACCATTGTTC	131
SsWRKY44	GCTCGTCATCACCTACACCTC	TTCCTCCATCTCCTCCTCCTT	193
SsWRKY47	CAACGACCGACGAGATGATAGT	AATCCTTCAACTCCAACCAGAGAT	128
SsWRKY49	CATCGGACGCTCACGGAAT	CGCCACCTGTAGCCATCAT	80
SsWRKY51	TCCAAATCAAGGGCAAGACAAAC	CCTCCTCCTCCACCTCCATA	137
SsWRKY53	CATACGAAGGCGAGCACAAC	CGGCGAGTCCAATCTTAGGT	200
SsWRKY56	AATGTGGAAGCAGTGGTAGATGT	TTATAGCAGGCACCTTGATGGAT	81
SsWRKY57	TCTTCCTTGGCTCCGAATGTT	TTCTGCTCCGTCTGCTCATC	140
